# Complete Genome Sequence of *Pediococcus pentosaceus* Strain wikim 20, Isolated from Korean Kimchi

**DOI:** 10.1128/genomeA.01233-16

**Published:** 2016-11-10

**Authors:** Se Hee Lee, Min Young Jung, Boyeon Park, Sohn Sung-Oh, Hae Woong Park, Hak-Jong Choi, Jong-Hee Lee

**Affiliations:** World Institute of Kimchi, Gwangju, Republic of Korea

## Abstract

*Pediococcus pentosaceus* strain wikim 20 is a lactic acid bacterium that was isolated from kimchi, a representative traditional Korean fermented food. Here, we announce the complete genome sequence of *P. pentosaceus* strain wikim 20 consisting of a 1,830,629-bp chromosome and provide a description of its annotation.

## GENOME ANNOUNCEMENT

Kimchi is a representative Korean fermented food, which is made by a variety of vegetables such as cabbage or radish with seasoning ingredients including red pepper powder, garlic, leek, ginger, and salt. It is produced through fermentation of salted vegetables by lactic acid bacteria at a low temperature (1–4).

*Pediococcus pentosaceus* strains of the phylum *Firmicutes* and the family *Lactobacillaceae* originally have been isolated from plant materials, processed meats, and vegetable pickles (5, 6). Some *Pediococcus pentosaceus* strains were known as effective bacteria, such as bacteriocin producers (7, 8) and potential probiotic bacteria (6).

The strain wikim 20 was isolated from cabbage kimchi. The genome of the strain wikim 20 was sequenced using the Pacific Biosciences RS II platform with a 20-kb SMRTbell template library at Macrogen (Seoul, Republic of Korea), which generated 1,137,930,148 bp (164,566 reads; about 175.5-fold genome coverage). The resulting sequences were assembled using the Hierarchical Genome Assembly Process version 3.0 (HGAP3.0). The assembled genome of *P. pentosaceus* wikim 20 is composed of one circular chromosomal genome of 1,739,283 bp (37.2% G+C content) and three circular plasmids. The complete genome sequence of strain wikim 20 was annotated using the NCBI Prokaryotic Genome Automatic Annotation Pipeline (PGAAP) (9) and the Rapid Annotations using Subsystem Technology (RAST) server version 2.0. The tRNA and rRNA genes were annotated using the tRNAscan-SE (10) and RNAmmer (11) software programs, respectively. Annotation of the genome sequence resulted in the determination of 1,779 predicted coding sequences, 55 tRNA genes, and 5 rRNA operons. The protein functions were grouped according to clusters of orthologous group (COG) on the BASys web server using Glimmer gene prediction (12, 13). Among the 1,779 predicted coding sequences, 1,529 were classified into 20 COG functional categories: C, D, E, F, G, H, I, J, K, L, M, N, O, P, Q, R, S, T, U, and V. Genome annotation revealed the presence of 159 genes involved in central carbohydrate, polysaccharide, fermentation, and sugar metabolism and 24 genes related to stress (low pH, temperature, and salt) resistance. These results maybe reflect the microorganism’s possible survival strategy in kimchi and other fermented food.

### Accession number(s).

The complete genome information for the chromosome of *Pediococcus pentosaceus* wikim 20 was deposited in NCBI/GenBank under the accession number CP015918, and the information for the plasmids pKPP1 to pKPP3 was deposited in GenBank under the accession numbers CP015919 to CP015921, respectively. *Pediococcus pentosaceus* wikim 20 KCTC 21076 is available from the Korean Collection for Type Cultures (KCTC, Jeongeup, Republic of Korea).
